# Effect of Behavioral Factors on Severity of Female Pattern Hair Loss: An Ordinal Logistic Regression Analysis

**DOI:** 10.7150/ijms.45979

**Published:** 2020-06-27

**Authors:** Yanhua Yi, Xiaoqiang Li, James Jia, Djakaya Ngondi Guy Didier, Jun Qiu, Jile Fu, Xiaoyan Mao, Yong Miao, Zhiqi Hu

**Affiliations:** Department of Plastic and Aesthetic Surgery, Nanfang Hospital of Southern Medical University, Guangzhou, Guangdong 510515, China.

**Keywords:** Female pattern hair loss (FPHL), alcohol consumption, alopecia, traction, China

## Abstract

**Background:** Female pattern hair loss (FPHL) is one of the most common types of hair loss with complex genetic predisposition. A frontal pattern hair loss with ponytail hairstyle is pervasively seen among young Chinese women. The purpose of this study is to investigate the association between the severity of FPHL and behavioral factors which include dietary, and sleep habits, and to test the hypothesis on whether ponytail hairstyle is an independent factor that increases the risks of being more severe on the FPHL scale.

**Methods:** A cross-sectional survey was performed with a structured questionnaire in this study. The severity of FPHL was graded according to basic and specific (BASP) classifications. Ordinal logistic regression analysis was performed to investigate the factors related to the severity of FPHL.

**Results:** 1,825 participants with different severities of FPHL completed the questionnaire. Ordinal logistic regression analysis revealed that the age group between thirty and forty years (OR:2.03, 95% CI: 1.56,2. 65), insufficient time with poor quality (OR:1.30, 95% CI: 1.05,1.62), presence of alcohol consumption (OR:2.15, 95% CI: 1.14,4.42), ponytail hairstyles (OR:2.03, 95% CI: 1.40,2.96), and oily scalps (OR:2.00, 95% CI: 1.65,2.43) were risk factors which increased the odds of being in the more severe type of FPHL, compared to the age group that ranged from eighteen to thirty years, sufficient sleep with good quality, without alcohol consumption, ponytail hairstyles, and oily scalps.

**Conclusion:** Avoiding alcohol consumption and ponytail hairstyles, in combination with proper control of scalp oil, improve sleep quality with sufficient time may help prevent FPHL from deteriorating to the more severe type.

## Introduction

Female pattern hair loss (FPHL) is the most common type of hair loss among women 1. As hair loss is less socially acceptable for women, FPHL usually has greater impact on women than it does in men. FPHL in Chinese women differs from Caucasian counterparts not only in its lower prevalence, but also in clinical manifestations 2-4. In addition to the Caucasian FPHL characteristics as a diffuse thinning of the hair on the crown 5, 6, FPHL in Chinese women often present with a frontal pattern hair loss 7.

The early onset of alopecia in recent years, which could not be accounted with genetic reasons, urges researchers to investigate behavioral factors, such as body concentration of heavy metals, smoking and other dietary risks 8. Similar to male pattern hair loss, there may be an association between FPHL and hypertension, insulin resistance, and coronary artery diseases 9-11. In spite of the increasing studies addressing different factors related to FPHL, there is a paucity of data investigating the association of behavioral factors with the severity of FPHL, especially among a big sampling young woman. This study is primary to investigate the association between the severity of FPHL and behavioral factors including carnivorous eating, vegetable intake, junk food, alcohol consumption, and stress in work, sleep habits, smoking, and scalp health management.

Traction alopecia is well documented among women wearing hair extensions 12. A study demonstrated the association between frontal pattern hair loss among Chinese women and cultural hair styling, but the sample size was small 13. we hypothesized that ponytail hairstyle would be a type of traction alopecia, which may be associated with the severity of alopecia. The second objective of this study is to test this hypothesis on whether ponytail hairstyle is an independent risk factor related to the severity of alopecia with a big study population.

## Materials and Methods

### Study Design and Participants

A web based cross-sectional survey was performed from September 2018 to September 2019 with a structured questionnaire through the Haotoufa Bulletin Board System (BBS) (https://bbs.haotoufa.com/), one of the most popular websites in China, which provides shared information for AGA groups with almost one million registered users. A panel of researchers including two epidemiologists, a statistician, four dermatologists, two plastic and aesthetic surgeons reviewed the questionnaire to ensure its validity. A small pilot study was conducted to verify the suitability of the questions.

Informed consent was obtained from all individual participants in the study. The protocol was approved by the Ethics Committee of Southern Medical University, China.

### Assessment

The severity of FPHL was graded according to basic and specific (BASP) classifications by participants first and double checked by two dermatologists 14. Referred to a previous study, we categorized the mild group as L, M0, C0, M1, C1, F1, V1, the moderate group as M2, C2, F2, V2, and the severe group as M3, C3, F3, V3, U1∼3 15. The independent variables were age group, marital status, working factors (mental work or physical work, work stress), lifestyle factors (smoking, alcohol consumption), dietary habits (carnivorous diet, vegetarian diet, junk food, sugary rinks), and scalp conditions. In order to test this hypothesis on whether ponytail hairstyle is an independent risk factor related to the severity of FPHL, the participants were asked whether or not they regularly wear ponytail hairstyles in daily life. Concerning carnivorous diets, respondents were assessed by asking whether they consume more meat than vegetables in their diets. The vegetarian diet was assessed by asking whether the respondents consume more vegetables than meat. Junk foods were defined and classified as those commercial products including candy, ice cream, salty snacks with plenty of calories, salt, and fats consumed at least twice a week. Sugary drinks, mainly sweetened soy bean milk and milk products, with an exception to coffee, coke and sweetened tea, were specified in the questionnaire. For lifestyle factors which are smoking and alcohol consumption, participants were asked whether they smoke and/or take alcohol currently in daily life.

### Statistical analysis

R software (R language version 3.5.2) was used for data analysis 14. Descriptive data were presented as frequencies and percentages. The chi-square test and Fisher's test were used as appropriate for categorical variables. Ordinal logistic regression analysis was performed to investigate the factors related to the severity of FPHL. A p-value<0.05 indicated statistical significance.

## Results

In order to investigate the factors related to the severity of FPHL among comparatively young Chinese women whose dietary and behavioral factors were more homogenous, the participants younger than forty years were included in the final analysis. In total, 2,717 female participants aged between eighteen and forty years with different levels of FPHL participated in the survey and 1,825 (69%) of them completed the questionnaire.

As shown in Table 1, among the total 1,825 participants, 1,321 (72.4%) were less than thirty years old, 504 (27.6%) were aged from thirty to forty years. In the mild group, the participants younger than thirty years took an apparent bigger percentage, whereas in moderate and severe groups, the percentages of participants aged from thirty to forty increased. The distribution among groups with different levels of severity by marital status, sleep, mental workload, ponytail hairstyle, alcohol consumption and scalp health were different using a univariate analysis. But the difference among groups with different severity by factors including outdoor physical labor, high stress in work, smoking, carnivorous eating, junk food consumption, weight control, sugary drinks, and vegetarian eating were not significant.

Table 2 made a preliminary ordinal logistic regression analysis on the factors associated with the severity of FPHL. The results showed that marital status, sleep, mental workload, high stress in work, ponytail hairstyle, alcohol consumption and scalp health were potential risk factors.

The factors with a p value less than 0.1 in the above preliminary analysis were included to make a further analysis with adjustment of age groups. Table 3 summarized the prime mode containing factors associated with the severity of FPHL. The results revealed that age groups, marital status, insufficient time with poor quality, ponytail hairstyle, alcohol consumption, oily scalp were associated with the severity of FPHL. The factors within the thirty to forty years age group (OR: 2.03, 95% CI: 1.56, 2.65), insufficient time with poor quality (OR: 1.30, 95% CI: 1.05, 1.62) , presence of alcohol consumption (OR: 2.15, 95% CI: 1.14, 4.42), ponytail hairstyles (OR: 2.03, 95% CI: 1.40, 2.96), and oily scalps (OR: 2.00, 95% CI: 1.65, 2.43) as risk factors, chances of developing the more severe type of FPHL were more apparent.

## Discussion

Based on a multi-center study, the prevalence of FPHL in Chinese women was much lower than that in Caucasians 2. Studies have shown that FPHL has genetic predispositions 16, 17, but it is hard to explain the tendency of the increasing prevalence of FPHL with decreasing age of onset in recent years 18. The findings that FPHL has an association with metabolic syndrome (Mets) and cardiovascular diseases in young women indicated that behavioral and dietary factors may contribute to the severity of FPHL 19.

Under lifestyle factors, alcohol consumption was associated to the severity of FPHL in the final ordinal regression analysis. After a control for the age groups and all other potential factors, the result was similar to one Korean research but contrary to an Italian study 20, 21. Smoking was found to increase the likelihood of having FPHL in previous study 20, after controlling potential factors, smoking was a very weak risk factor (OR:1.59, 95% CI: 0.97,2.57) in this study. A Taiwan study suggested that soy bean drink consumption would provide protective effects against alopecia, but this study did not support that 8.

In the clinical setting, we found some common points among young Chinese women with FPHL, which include similar working style with high stress and late sleep behaviors. Therefore, we hypothesized that working styles and work-related stress were possible risks to the severity of FPHL. However, this study could not get any supporting evidence. The findings that insufficient time with poor quality associated with the severity of FPHL had important significance. Another interesting common point was that a frontal pattern hair loss is commonly observed among Chinese women with FPHL, especially with ponytail hairstyles. We hypothesized that ponytail hairstyles may increase tension on the scalp, and then lead to alopecia. This study tested the hypothesis, and found that the young women with ponytail hairstyles would have a 2.04 times higher risk of developing a more severe grade of FPHL than those without this hairstyle.

Oily scalp is a common symptom of Seborrheic Dermatitis. We found out that it was related to the severity of FPHL, coinciding with a previous Taiwan study 8. It is imperative to take proper treatment of oily scalps, which would be helpful in preventing developing into the more severe type.

In conclusion, avoiding alcohol consumption and ponytail hairstyles, as well as exercising proper control of oily scalps, improve sleep quality with sufficient time would help prevent FPHL from deteriorating into the more severe type. This would be beneficial for both patient education and clinical consultation.

## Limitations and future directions

Although this study firstly revealed the association between alcohol consumption, ponytail hairstyle, sleep quality and severity of FPHL among Chinese women, there were some limitations needed for further studies. Firstly, the frequency and volume of alcohol consumption was not clearly defined in the current study. Moreover, the mechanism on why alcohol consumption increased chances of being more severe alopecia remained unknown. Secondly, this study tested the hypothesis that the ponytail hairstyle would be an independent factor increasing the severity of FPHL especially in those women presenting with frontal pattern hair loss, but however, whether a change of the hairstyle would prevent or even reverse alopecia conditions for FPHL needs further investigations.

Despite these limitations, this study made important contributions to give data-based evidence on consultation and patient education in clinical setting.

## Figures and Tables

**Table 1:** Female pattern hair loss by age group, marital status, sleep, work and personal behaviors, dietary factors, and scalp health

Variables	Mild (881)	Moderate (709)	Severe (235)	P value
**Age group, years**				< 0.001
18-30	712 (80.8)	484 (68.3)	125 (53.2)	
30-40	169 (19.2)	225 (31.7)	110 (46.8)	
**Marital Status**				< 0.001
Single	568 (64.5)	350 (49.4)	100 (42.6)	
Married without Childbearing	84 (9.5)	102 (14.4)	23 (9.8)	
Married with Childbearing	229 (26)	257 (36.2)	112 (47.7)	
**Sleep**				< 0.001
Sufficient time with good quality	310 (35.2)	211 (29.8)	58 (24.7)	
Insufficient time with good quality	283 (32.1)	210 (29.6)	70 (29.8)	
Insufficient time with poor quality	288 (32.7)	288 (40.6)	107 (45.5)	
**Work and behavior factors**				
Mental work load				0.009
No	491 (55.7)	418 (59)	157 (66.8)	
Yes	390 (44.3)	291 (41)	78 (33.2)	
Outdoor physical labor				0.129
No	850 (96.5)	675 (95.2)	220 (93.6)	
Yes	31 (3.5)	34 (4.8)	15 (6.4)	
High stress in work				0.111
No	408 (46.3)	322 (45.4)	91 (38.7)	
Yes	473 (53.7)	387 (54.6)	144 (61.3)	
Smoking				0.177
No	857 (97.3)	683 (96.3)	223 (94.9)	
Yes	24 (2.7)	26 (3.7)	12 (5.1)	
Ponytail hairstyle				< 0.001
No	840 (95.3)	668 (94.2)	205 (87.2)	
Yes	41 (4.7)	41 (5.8)	30 (12.8)	
**Dietary factors**				
Alcohol consumption				0.02
No	873 (99.1)	697 (98.3)	227 (96.6)	
Yes	8 (0.9)	12 (1.7)	8 (3.4)	
Carnivorous eating				0.791
No	741 (84.1)	589 (83.1)	199 (84.7)	
Yes	140 (15.9)	120 (16.9)	36 (15.3)	
Junk food				0.197
No	633 (71.9)	490 (69.1)	176 (74.9)	
Yes	248 (28.1)	219 (30.9)	59 (25.1)	
Weight control				0.171
No	747 (84.8)	600 (84.6)	210 (89.4)	
Yes	134 (15.2)	109 (15.4)	25 (10.6)	
Sugary drinks				0.429
No	703 (79.8)	548 (77.3)	188 (80)	
Yes	178 (20.2)	161 (22.7)	47 (20)	
Vegetarian eating				0.382
No	759 (86.2)	621 (87.6)	210 (89.4)	
Yes	122 (13.8)	88 (12.4)	25 (10.6)	
**Scalp health**				
Dandruff				0.002
No	689 (78.2)	581 (81.9)	207 (88.1)	
Yes	192 (21.8)	128 (18.1)	28 (11.9)	
Itchy scalp				0.144
No	565 (64.1)	424 (59.8)	153 (65.1)	
Yes	316 (35.9)	285 (40.2)	82 (34.9)	
Oily scalp				< 0.001
No	369 (41.9)	202 (28.5)	57 (24.3)	
Yes	512 (58.1)	507 (71.5)	178 (75.7)	

Numbers in bracket are percent unless otherwise stated.

**Table 2:** Preliminary ordinal logistic regression analysis of factors that associated with the severity of female pattern hair loss

Variables	Explanation of variables	Ordinal odds ratio (95%CI)	P value
Marital status	Single	1.00 (reference)	
	Married without Childbearing	1.67 (1.27,2.21)	<0.001
	Married with Childbearing	2.08 (1.71,2.52)	<0.001
Sleep	Sufficient time with good quality	1.00 (reference)	
	Late sleep with insufficient time	0.86 (0.69,1.08)	0.09
	Insufficient time with poor quality	1.37 (1.11,1.70)	0.002
Work and behavior factors	**Mental work load**		
	No	1.00 (reference)	
	Yes	0.81 (0.67,0.96)	<0.01
	**Outdoor physical labor**		
	No	1.00 (reference)	
	Yes	1.33 (0.86,2.04)	0.1
	**High stress in work**		
	No	1.00 (reference)	
	Yes	1.17 (0.98,1.40)	0.04
	**Smoking**		
	No	1.00 (reference)	
	Yes	1.43 (0.89,2.31)	0.07
	**Ponytail hairstyle**		
	No	1.00 (reference)	
	Yes	1.92 (1.3,2.80)	<0.01
Dietary factors	**Alcohol consumption**		
	No	1.00 (reference)	
	Yes	2.54 (1.25,5.16)	<0.01
	**Carnivorous eating**		
	No	1.00 (reference)	
	Yes	0.98 (0.77,1.26)	0.44
	**Junk food**		
	No	1.00 (reference)	
	Yes	0.98 (0.80,1.20)	0.43
	**Weight control**		
	No	1.00 (reference)	
	Yes	0.86 (0.67,1.10)	0.11
	**Sugary drinks**		
	No	1.00 (reference)	
	Yes	1.10 (0.88,1.37)	0.21
	**Vegetarian eating**		
	No	1.00 (reference)	
	Yes	0.85 (0.65,1.11)	0.12
Scalp health	**Oily scalp**		
	No	1.00 (reference)	
	Yes	1.87 (1.55,2.26)	<0.001

Ordinal Logistic Regression analysis.

**Table 3:** Ordinal logistic regression analysis of factors that associated with the severity of female pattern hair loss with adjustment of age groups

Variables	Explanation of variables	Ordinal odds ratio (95%CI)	P value
Age, years	18-30	1.00 (reference)	
	31-40	2.03 (1.56,2.65)	< 0.001
Sleep	Sufficient time with good quality	1 (reference)	
	Late sleep with insufficient time	0.98 (0.78,1.24)	0.46
	Insufficient time with poor quality	1.30 (1.05,1.62)	0.01
Work and behavior factors	**Ponytail hairstyle**		
	No	1.00 (reference)	
	Yes	2.03 (1.40,2.96)	<0.001
	**Mental work load**		
	No	1.00 (reference)	
	Yes	0.87 (0.73,1.05)	0.08
Dietary factors	**Alcohol consumption**		
	No	1.00 (reference)	
	Yes	2.15 (1.14,4.42)	0.01
Scalp health	**Oily scalp**		
	No	1.00 (reference)	
	Yes	2.00 (1.65,2.43)	<0.001

Ordinal Logistic Regression analysis.
